# Hesperidin - loaded PVA/alginate hydrogel: targeting NFκB/iNOS/COX-2/TNF-α inflammatory signaling pathway

**DOI:** 10.3389/fimmu.2024.1347420

**Published:** 2024-04-15

**Authors:** Ahmad S. Kodous, Mostafa A. Abdel-Maksoud, Mohamed A. El-Tayeb, Diana A. Al-Sherif, Suzan Shawky Abuelkasem Mohamed, Mohamed Mohamady Ghobashy, Ayat M. Emad, Shady M. Abd El‐Halim, Soheir A. A. Hagras, Samson Mani, Arunagiri Kuha Deva Magendhra Rao, Ahmed M. Hussein, Helen N. Saada

**Affiliations:** ^1^ Department of Molecular Oncology, Cancer Institute Women's Indian Association (WIA), Tamilnadu, India; ^2^ Radiation Biology Department, National Center for Radiation Research and Technology (NCRRT), Egyptian Atomic Energy Authority (EAEA), Cairo, Egypt; ^3^ Botany and Microbiology Department, College of Science, King Saud University, Riyadh, Saudi Arabia; ^4^ Applied Medical Science Faculty, Sixth October University, Sixth of October City, Egypt; ^5^ Biochemistry and nutrition Department, Faculty of Applied Health Science Technology, Sixth October University, Sixth of October City, Egypt; ^6^ Radiation Research of Polymer Chemistry Department, National Center for Radiation Research and Technology, Egyptian Atomic Energy Authority, Cairo, Egypt; ^7^ Pharmacognosy Department, Faculty of Pharmacy, October 6 University, Sixth of October City, Giza, Egypt; ^8^ Department of Pharmaceutics and Industrial Pharmacy, Faculty of Pharmacy, October 6 University, Sixth of October City, Giza, Egypt; ^9^ Department of Drug Radiation Research, National Center for Radiation Research and Technology (NCRRT), Egyptian Atomic Energy Authority, Cairo, Egypt; ^10^ Zoology Department, Faculty of Science, Al Azhar University, Assiut, Egypt; ^11^ Department of Pharmaceutical Sciences, Division of Pharmacology and Toxicology, University of Vienna, Vienna, Austria

**Keywords:** hydrogel, hesperidin, wound healing, wound dressing, biomaterial hydrogel, gamma irradiation

## Abstract

**Introduction:**

Skin injuries represent a prevalent form of physical trauma, necessitating effective therapeutic strategies to expedite the wound healing process. Hesperidin, a bioflavonoid naturally occurring in citrus fruits, exhibits a range of pharmacological attributes, including antimicrobial, antioxidant, anti-inflammatory, anticoagulant, and analgesic properties. The main objective of the study was to formulate a hydrogel with the intention of addressing skin conditions, particularly wound healing.

**Methods:**

This research introduces a methodology for the fabrication of a membrane composed of a Polyvinyl alcohol - Sodium Alginate (PVA/A) blend, along with the inclusion of an anti-inflammatory agent, Hesperidin (H), which exhibits promising wound healing capabilities. A uniform layer of a homogeneous solution comprising PVA/A was cast. The process of crosslinking and the enhancement of hydrogel characteristics were achieved through the application of gamma irradiation at a dosage of 30 kGy. The membrane was immersed in a Hesperidin (H) solution, facilitating the permeation and absorption of the drug. The resultant system is designed to deliver H in a controlled and sustained manner, which is crucial for promoting efficient wound healing. The obtained PVA/AH hydrogel was evaluated for cytotoxicity, antioxidant and free radical scavenging activities, anti-inflammatory and membrane stability effect. In addition, its action on oxidative stress, and inflammatory markers was evaluated on BJ-1 human normal skin cell line.

**Results and Discussion:**

We determined the effect of radical scavenging activity PVA/A (49 %) and PVA/AH (87%), the inhibition of Human red blood cell membrane hemolysis by PVA/AH (81.97 and 84.34 %), hypotonicity (83.68 and 76.48 %) and protein denaturation (83.17 and 85.8 %) as compared to 250 μg/ml diclofenac (Dic.) and aspirin (Asp.), respectively. Furthermore, gene expression analysis revealed an increased expression of genes associated with anti-oxidant and anti-inflammatory properties and downregulated TNFα, NFκB, iNOS, and COX2 by 67, 52, 58 and 60%, respectively, by PVA/AH hydrogel compared to LPS-stimulated BJ-1 cells. The advantages associated with Hesperidin can be ascribed to its antioxidant and anti-inflammatory attributes. The incorporation of Hesperidin into hydrogels offers promise for the development of a novel, secure, and efficient strategy for wound healing. This innovative approach holds potential as a solution for wound healing, capitalizing on the collaborative qualities of PVA/AH and gamma irradiation, which can be combined to establish a drug delivery platform for Hesperidin.

## 1 Introduction

Skin injuries are consistently recognized as prevalent physical traumas. Individuals with diabetes or those undergoing radiotherapy often experience challenges related to chronic skin wounds and impaired wound healing. Given the diverse nature of skin injuries, an array of products has been developed to address and treat varying types of skin lesions (1). Recent advancements in wound dressings have emphasized the importance of localized drug delivery for wound healing, driven by concerns regarding the adverse effects associated with systemic drug delivery (2, 3). Numerous novel dressings have been created with the goal of enhancing the natural wound healing process. The optimal wound dressing should establish a moist environment, shield the wound from potential infections, and expedite the wound healing process (4).

Wound healing is a complex process characterized by clot formation, inflammation, proliferation and remodeling. When blood vessels are injured, the body stops the blood flow (haemostasis), activates platelets to allow clot formation and releases growth factors, which starts the healing process (5, 6). This is followed by an inflammatory response. During the initial stages of the inflammatory response, neutrophils release active antimicrobial agents and proteases, initiating the process of eliminating damaged tissue. In the later stages of the inflammatory response, typically around three days following the injury, monocytes undergo maturation into macrophages. These macrophages play a crucial role in phagocytizing bacteria, deceased neutrophils, and damaged tissue (7). Furthermore, macrophages also secrete growth factors, chemokines, and cytokines, which subsequently induce the migration and proliferation of keratinocytes, fibroblasts, and endothelial cells (8). Additionally, macrophages contribute to the process of angiogenesis and play a role in the formation of vascularized connective tissue, ultimately resulting in the closure of the wound (9). The remodeling phase commences in the proliferation stage and extends over an extended duration. In this stage, the body engages in both collagen production and its breakdown, ensuring a delicate equilibrium between the requirement for tensile strength and the remodeling of new tissue (9). Hence, owing to their inherent physical attributes, including swelling and biodegradability, hydrogels encompass many of the sought-after qualities of an ideal wound dressing (6, 10). A recent study by Bagher et al. (10) was shown that hydrogels composed of alginate and chitosan, and incorporating the flavonoid hesperidin, have the potential to expedite the wound healing process. This acceleration is attributed to their attributes, including controlled drug release, antibacterial properties, and biocompatibility. Hesperidin, a compound naturally found in citrus fruits, exhibits a diverse range of pharmacological properties, including antimicrobial, antioxidant, anti-inflammatory, angiogenic, anticoagulant, and analgesic characteristics. These attributes suggest its potential utility as a therapeutic agent in the context of skin diseases (10). Studies have reported that hesperidin functions as a potent antioxidant (11, 12), also it has been demonstrated to possess anti-inflammatory properties and to act as a scavenger of free radicals. Hesperidin plays a vital role in supporting collagen retention, and it is integral for the effective absorption and utilization of vitamin C (13). Studies have shown that Hesperidin is non-toxic in both animals and humans, and it has been observed to reduce the duration of healing for radiation-induced wounds (14–16). The 10% hesperidin hydrogel exhibited the most favorable wound healing outcomes in animal studies. Erol et al. (17, 18) formulated novel hydrogels using polyvinyl alcohol, incorporating zinc oxide nanoparticles loaded with hesperidin. Their investigation encompassed an examination of the biological and thermal characteristics of these hydrogels (10). The authors loaded hesperidin onto zinc oxide nanoparticles through the utilization of the hydrothermal method. This process facilitated the uniform dispersion of zinc oxide nanoparticles within the polyvinyl alcohol matrix, resulting in a significant enhancement of its thermal stability and a reduction in its softening temperature. Additionally, the hydrogels acquired valuable antibacterial attributes from the zinc oxide-hesperidin compound. They demonstrated antibacterial efficacy against both E. coli and Staphylococcus aureus. Moreover, the antioxidant capacity of the polyvinyl alcohol increased in proportion to higher concentrations of zinc oxide-hesperidin. Importantly, the hydrogels displayed no cytotoxic effects on human cancer cells and did not stimulate cell proliferation (17). The zinc oxide-hesperidin nanoparticles led to a decrease in the water solubility and water holding capacity of the unadulterated polyvinyl alcohol. Furthermore, the hydrogels exhibited notable enhancements in thermal resistance when compared to the pure polyvinyl alcohol (17). Hydrogels are naturally present in the form of polymer networks like collagen or gelatin, and they can also be produced synthetically. Polyvinyl alcohol (PVA), an artificial hydrophilic polymer, is characterized by its non-toxic, biocompatible, and biodegradable properties (19, 20). PVA-based hydrogels featuring three-dimensional network structures that result from crosslinking and swelling processes. Alginate, an anionic polymer found in nature, is widely employed in various biomedical applications owing to its biocompatibility, low toxicity, and ease of gelation. Alginate hydrogels are typically formulated through cross-linking techniques. Their structural resemblance to the extracellular matrices found in living tissues makes them suitable for applications in wound healing and drug delivery (21). Alginate wound dressings are recognized for their ability to sustain a physiologically moist microenvironment, reduce the risk of bacterial infection at the wound site, and contribute to the facilitation of wound healing (22).

In the present investigation, a hydrogel was formulated using polyvinyl alcohol and sodium alginate, with the aid of gamma radiation technology. Subsequently, this hydrogel was loaded with hesperidin, and a comprehensive evaluation was conducted to assess its cytotoxicity, antioxidant and free radical scavenging properties, anti-inflammatory characteristics, and membrane stability effects. Additionally, the impact of this hydrogel on oxidative stress and inflammatory markers was examined using the BJ-1 human normal skin cell line.

## 2 Materials and methods

### 2.1 Chemicals and devices

The Polyvinyl Alcohol (PVA) used in the study was obtained from Loba Chemie Pvt. Ltd., Mumbai, India. This particular PVA had a degree of polymerization ranging from 1700 to 1800 and a molecular weight of 115,000 g·mol^−1^. Additionally, Alginate (A) had average molecular weight of 120000 g·mol−1 and Hesperidin (H) were obtained from Sigma-Aldrich, St Louis, USA.

### 2.2 Radiation synthesis of PVA/A loaded with hesperidin hydrogel

The procedure for preparing PVA/AH is outlined as follows: Dissolve 10 grams of Polyvinyl Alcohol (PVA) in 100 mL of deionized water. Stir the mixture continuously at a temperature of 70°C until a homogeneous solution is obtained. Dissolve 5 grams of Sodium Alginate (A) in the 50 ml of PVA solution containing the dissolved PVA. Mix the solution thoroughly to ensure proper dispersion. The mixture should be allowed to cool to the ambient temperature. After the liquid has achieved ambient temperature, proceed to pour it into a Petri plate (diameter 15 cm × height 1.5 cm). The casting process involves spreading the mixture evenly across the dish’s surface. Allow the casted mixture to dry naturally at room temperature. This step involves evaporating the solvent (water) from the mixture, resulting in the formation of a solid membrane. Subject the dried membrane to gamma irradiation with a dose of 30 kGy (0.8 kGy/h) to modify and sterilize materials. Dissolve 400 mg of Hesperidin in 5 ml of water to create a Hesperidin solution. Immerse the obtained PVA/A blend membrane (ratio 50:50 w/w) into the Hesperidin solution in a Petri dish. Allow the membrane to soak in the Hesperidin solution, facilitating the loading of Hesperidin into the membrane. The result of this procedure is a PVA/A blend membrane infused with Hesperidin. The process allows the creation of a membrane PVA/AH that combines the properties of PVA and Sodium Alginate with the presence of Hesperidin, potentially for controlled release or drug delivery systems.

### 2.3 FTIR analysis

For the investigation of compatibility and interactions between PVA and Hesperidin (H) molecules, FTIR analysis was performed. Spectra were acquired using a Shimadzu IRAffinity-1 spectrometer, equipped with a single-reflection Attenuated Total Reflectance (ATR) attachment series microscope from Shimadzu, Japan. The spectral data were recorded within the wavenumber range spanning from 400 cm^-1^ to 4000 cm^-1^. A resolution of 4 cm^-1 was employed to ensure high-resolution spectral information. Each spectrum was an average of 32 scans, enhancing the signal-to-noise ratio and bolstering the reliability of the data collected. To ensure the quality of the FTIR spectra, a baseline correction was applied during data processing. This correction effectively eliminated any background variations, resulting in precise and uncontaminated spectral data. Additionally, the FTIR spectra were presented in the absorbance scale, providing a direct representation of the measured data. This approach offers a more accurate depiction of the interactions between PVA and Hesperidin. Moreover, for the validation of the successful incorporation of hesperidin into the polymer matrix, FTIR spectra of the hydrogel mixture with H and H in isolation were presented. This comparative analysis distinctly reveals the interactions and chemical bonds formed when H is combined with the PVA matrix, substantiating its effective integration. These clarifications provide a comprehensive description of the FTIR analysis, encompassing specific details related to the wavenumber range, resolution, scans, data processing, and the presentation of spectra for H in isolation and within the hydrogel matrix to underscore its incorporation.

### 2.4 Water vapor transmission rate measurement

The Water Vapor Transmission Rate (WVTR) was assessed following the ASTM method E96-90, Procedure D. To ensure controlled conditions and minimize ambient influences, an evaporimeter was developed within a sealed glass chamber. The setup consisted of a glass chamber with a lid, an isothermal bath fixed at 35°C, a digital hygrometer providing continuous readings for % relative humidity (RH), temperature, and dew point, and a reservoir holding a saturated magnesium chloride solution. A permeability cup, with a size of 5* cm* x 1.5* cm* and a volume of around 30* ml*, was filled with 20 grams of deionized distilled water. The test membrane (PVA/A) was securely attached over the cup’s opening. The evaporation of water through the test membrane was continuously observed by measuring the decrease in weight of the cup over a period of time. An open cup was employed as a control to establish a reference point for comparison. The relative humidity (RH) within the evaporimeter stabilized at approximately 40%.

### 2.5 Oxygen gas flux measurement

The determination of oxygen gas flux through the membranes was carried out using the Yanaco GTR-10 Gas Permeation Analyzer (GPA) (Yanagimoto Co., Kyoto, Japan) while maintaining a constant temperature of 35°C. The (PVA/A) membranes were positioned within a diffusion cell with a transmission area of approximately 3.14 cm^2^. Dried air was introduced into one side of the membrane, and the other side was subjected to a vacuum. The flux of oxygen that permeated the membrane was quantified using a gas chromatography analyzer.

### 2.6 Water absorption measurement

The water absorption capacity of the (PVA/A) wound dressing was assessed by immersing a known weight (200 mg) of the dressing in a phosphate-buffered saline (PBS) solution. Once equilibrium in absorption was attained, the wet weight of the wound dressing was determined. Before measurement, the dressing was gently blotted with filter paper to eliminate any surface-bound water. The percentage of water absorption for the bilayer dressing was subsequently computed by comparing the initial weight to the augmented weight of the dressing.

### 2.7 Gamma irradiation cell

Gamma irradiation in this study was carried out utilizing a cobalt-60 gamma cell facility situated at the National Center for Radiation Research and Technology (NCRRT), a division of the Atomic Energy Authority of Egypt (AEAE) in Cairo. The cobalt^-60^ gamma cell employed in the irradiation process was produced in India and operated at a dose rate of 0.8 kiloGray per hour (kGy/h) (23, 24).

### 2.8 Preparation of buffer solution of different pH’s

The investigation encompassed the assessment of sample swelling under various pH conditions using different solutions as shown in **Table 1**.

**Table 1:** The process of preparing solutions with varying pH.

pH Solution	Components	Preparation Method
**pH 1**	0.1 M HCl + 0.1 M KCl	Mix 3 ml of KCl with 97 ml of HCl, dilute to 100 ml
**pH 2**	0.1 M HCl + 0.1 M KCl	Mix 89.4 ml of KCl with 10.6 ml of HCl, dilute to 100 ml
**pH 3**	0.1 M Citric Acid + 0.1 M Sodium Citrate dihydrate	Mix 46.5 ml of citric acid with 3.5 ml of sodium citrate, dilute to 100 ml
**pH 4**	0.1 M Citric Acid + 0.1 M Sodium Citrate dihydrate	Mix 31 ml of citric acid with 19 ml of sodium citrate, dilute to 100 ml
**pH 5**	0.1 M Citric Acid + 0.1 M Sodium Citrate dihydrate	Mix 20.5 ml of citric acid with 29.5 ml of sodium citrate, dilute to 100 ml
**pH 6**	0.1 M Citric Acid + 0.1 M Sodium Citrate dihydrate	Mix 7 ml of citric acid with 43 ml of sodium citrate, dilute to 100 ml
**pH 7**	0.1 M Disodium Hydrogen Phosphate + 0.1 M HCl + 0.1 M NaOH	Mix chemicals in a volume ratio of 75.6:24.4:0, dilute to 100 ml

### 2.9 Mechanical tensile properties

The tensile qualities of the dressing specimens were assessed with an Instron universal tester. The specimens were created with measurements of 15* cm* in length and 5* cm* in breadth. The selection of the machine orientation for the dressings was made in order to maintain uniform testing conditions throughout specimen extraction. The specimens that had been prepared were firmly affixed inside the grips of the Instron universal tester, which had a gauge length of 100* mm*. The rate of extension used throughout the testing procedure was established at 300 millimeters per minute. The rate of extension refers to the velocity at which the grips separate, resulting in the application of tensile forces on the specimen.

In order to guarantee the reliability and accuracy of the data, a total of five measurements were conducted for each variant of dressing. The recorded data included the measured values for criteria such as tensile strength, breaking extension, and other pertinent factors in each individual measurement. Ultimately, the mean values were determined by computing the average of the outcomes acquired from the five assessments. The research sought to acquire statistically meaningful and representative data on the tensile characteristics of the dressing specimens by the implementation of numerous measurements and subsequent calculation of mean values. This methodology aids in mitigating the possible effects of changes or fluctuations in the measured values, hence enhancing the dependability of the assessment of the tensile behavior of the dressing.

### 2.10 MTT cytotoxicity assay of PVA/A and PVA/AH hydrogels

The MTT test, also known as the 3-(4,5-dimethylthiazol-2-yl)-2,5-diphenyltetrazolium bromide test, is a reliable and expeditious technique. Human Normal Skin cell line (BJ-1) cultures were sourced from the Cell Culture Laboratory at VACSERA Company, located at 51 Ministry of Agriculture, Al Agouzah, Dokki, Giza Governorate, Egypt., Cells at a density of (1× 10^5^ per well) were incubated in a 5% CO_2_ environment for 48 hours, during which they were exposed to varying concentrations of PVA/A and PVA/AH ranging from 0 to 100 µg/ml. In order to commence the staining process, the standard MTT solution was diluted to produce a final concentration of 0.5 milligram MTT/ml. Following that, a volume of 300 µl of the MTT solution that had been decreased was introduced into each well that contained the cells. Following centrifugation of the cells at a speed of 15,000 rpm for a duration of 5 minutes, the culture media was removed by aspiration, and subsequently, the cells were consolidated into a pellet. Subsequently, a volume of 500 µl of a mixture containing isopropanol and hydrochloric acid was introduced, and the containers were aggressively agitated using a vortex mixer to ensure proper dispersion of the formazan crystals. Subsequently, the mixture underwent centrifugation at a speed of 15,000 rpm for a duration of 5 minutes in order to achieve homogenization. The spectrophotometer (Helios, UV/Visible, Leicestershire, UK) was used to measure the absorbance of the supernatants at a wavelength of 560 nm. To establish control, a solution comprising isopropanol and hydrochloric acid was employed, and its measurement was deducted from the recorded values of the other samples. The Equation (1) was employed to calculate the percentage of cell viability (25–28):


(1) % cell viability = [(Ac − Ab)/(As−Ac)] × 100

Where: absorbance_control_ (Ac), absorbance_blank_ (Ab), absorbance_sample_ (As).

### 2.11 DPPH radical scavenging activity of PVA/A and PVA/AH hydrogels

In brief, each 2.5-cm-diameter spherical specimen was subjected to a 10 ml methanol extraction, followed by one hour of agitation in a water bath. An aliquot of the material was then diluted with 10 ml of methanol. The reaction mixture, composed of sample (1.0 ml) and DPPH (3.0 ml) 3.0 ml of the DPPH with 1.0 ml of the sample, which had a concentration of 0.1 mM in methanol. The absorbance of the reaction mixture was measured after a duration of 30 minutes, with precautions taken to protect it from exposure to light. The solution was allowed to undergo incubation for a duration of thirty minutes at ambient temperature under dark conditions. Subsequently, the 517 nm absorbance was read. The calculation was performed using the Equation (2) (29–31).


(2) % scavenging = [(Ab − As)/Ab] × 100

### 2.12 Anti-inflammatory activity of PVA/A and PVA/AH hydrogels

The anti-inflammatory effects of the PVA/A and PVA/AH hydrogels were assessed through the following *in vitro* assays: a- Heat-induced hemolysis of human red blood cell (HRBC) membrane stabilization, b-hypotonicity–induced hemolysis, and c- protein (Egg albumin) denaturation (30, 31).

#### 2.12.1 Heat-induced hemolysis of HRBC and membrane stabilization

The hydrogel’s *in vitro* anti-inflammatory impact was assessed using HRBC membrane stabilization. As standards, aspirin (Asp) and diclofenac (Dic) were employed, and the percentage of RBC lysis was used as a measure of anti-inflammatory activity. It’s important to note that lysosomal membranes and HRBC membranes function similarly. If HRBC membrane stabilization occurs, the lysosomal membrane will likely stabilize as well. Read 560 nm absorbance. Blood samples were obtained from healthy volunteers, with exclusion criteria including the use of non-steroidal anti-inflammatory drugs (NSAID) and steroidal anti-inflammatory drugs (SAID) in the two weeks preceding the trial. Heparin was used to prevent blood clotting. All blood samples were stored at 4°C for 24 hours before use. After centrifugation for 5 minutes at 3000 rpm, the supernatant was removed. The cleaning process involved washing with sterile saline solution (0.9% w/v NaCl), subsequently, a further centrifugation was conducted for a duration of 5 minutes at a rotational speed of 3000 rpm. The determination of the packed cell volume was conducted following the repetition of the supernatant cleaning process on three occasions. A suspension with a concentration of 40% (v/v) was prepared by combining it with phosphate-buffered saline (10 mM, pH 7.4) in order to incorporate biological components (30, 31).

#### 2.12.2 Hypotonicity-induced hemolysis of HRBC

The experiment created an isotonic solution by mixing 154 mM NaCl with 10 mM sodium phosphite at pH 7.4. The control sample (Diclofenac or Aspirin) was mixed with distilled water, while the stock RBC suspension was mixed with 50 μl of distilled water, creating a hypotonic solution. After ten minutes at room temperature, the mixture was centrifuged at 5000 rpm for five minutes. A UV spectrophotometer assessed supernatant absorbance at 540 nm. Reference solutions included 250 µg/ml diclofenac and 250 µg/ml aspirin (30). Equation (3) calculated hemolysis inhibition percentage:


(3) $$\%\;Inhibition\;of\;hemolysis\;=100x\;[1-\left[\;\frac{\left(OD2-OD1\right)}{\left(OD3-OD1\right)}\right]\;$$

The notation used for the absorbance values in the experiment is as follows OD1: Test sample absorbance in isotonic solution, OD2: Test sample absorbance in hypotonic solution, OD3: Control sample absorbance in hypotonic solution.

#### 2.12.3 Protein denaturation test

The production of autoantigens can arise as a consequence of *in vivo* protein denaturation. Consequently, compounds possessing the capacity to impede protein denaturation may be harnessed for the development of anti-inflammatory pharmaceutical agents. The anti-inflammatory activity of PVA/A and PVA/AH hydrogels was evaluated by the % inhibition of heat-induced protein denaturation of egg albumin model. Hydrogel samples, 0.2 ml egg albumin, 2.8 ml PBS (pH 6.4), and saline were in the 5 ml reaction mixture. For control, the same volume of distilled water was used. After 15 minutes at 37.2°C, 5 minutes were heated at 70°C. At 660 nm, absorbance was measured. Similar absorbance of Asp 250 µg/ml and diclofenac 250 µg/ml were used for reference., as mentioned in Equation (4) (30–32).


(4) $$\%\;Inhibition\;of\;haemolysis\;=100x\;[1-\left[\;\frac{\left(OD2-OD1\right)}{\left(OD3-OD1\right)}\right]\;$$

The notation used for the absorbance values in the experiment is as follows OD1: Absorbance of test sample unheated, OD2: Absorbance of test sample heated, OD3: Absorbance of control sample heated.

### 2.13 Levels of ROS, NO, and MPO

To quantify the level of reactive oxygen species (ROS), the study employed the diacetyl dichlorofluorescein (DCFH-DA) 5M fluorescence probe and a standard curve for hydrogen peroxide (H_2_O_2_). The procedure involved combining an equal volume of cells diluted two-fold and DCFH-DA diluted by a factor of 1000, followed by an incubation period of 5 minutes at 37°C in the absence of light. Next, fluorescence emission was measured at 520 nm. This method measured sample ROS (33). The Griess assay measured NO at 490 nm. Griess reagent naphthyl ethylene-diamine dihydrochloride (0.1%), phosphoric acid (2%), and sulfanilamide (1%), was used to treat the cells. This reagent was used in conjunction with sodium nitroprusside to generate a bright-reddish-purple azo dye. The intensity of the color developed in this reaction was measured at 490 nm and served as an indicator of the NO level in the sample (34). In the measurement of myeloperoxidase (MPO) activity, both 1.2% H_2_O_2_ and 16.7 mg% ODD, were added with measurement unit μmoL of H_2_O_2_/min (35).

### 2.14 Determination the activity of GPX, SOD, and TAC

The activity of the antioxidant enzymes superoxide dismutase (SOD) and glutathione peroxidase (GPX) was evaluated using the pyrogallol autooxidation technique (36, 37).

The total antioxidant capacity (TAC) levels were assessed by mixing 2 mL of ABTS^•+^ a radical solution (produced by reacting ABTS with potassium persulfate; 1: 0.5) with 20 μL of the sample, in addition to using phosphate-buffered saline (PBS) as a control or butylated hydroxyanisole (BHT) as a reference standard. The ABTS [2,2’-azino-bis (3-ethylbenzothiazoline-6-sulfonic acid)] radical cation technique was employed. By utilizing the BHT standard curve, the TAC of the sample was determined as the BHT equivalent. This calculation was made after assessing the percentage of inhibition, which reflects the sample’s capacity to inhibit the oxidation of the ABTS radical cation (38).

### 2.15 Evaluation of gene expression of the inflammatory markers, TNF-α, NF-κB, iNOS, and COX-2

The degree of gene expression for nuclear factor-kappa B (NF-кB), inducible nitric oxide synthase (iNOS), cyclooxygenase-2 (COX-2), and tumor necrosis factor-alpha (TNF-α) was determined using the quantitative real-time reverse transcription-polymerase chain reaction (qRT-PCR) technique. This method allows for precise quantification of gene expression levels, providing valuable insights into the relative expression of these genes under specific conditions or treatments. In the process of RNA analysis, the GeneJET RNA Purification Kit was employed for the extraction of total RNA from the samples. To isolate the supernatant for RNA extraction, the cell lysate was subjected to centrifugation for 5 minutes at a speed of 14,000 revolutions per minute (rpm). Following this, the extracted RNA was quantified and then used for complementary DNA (cDNA) synthesis. Subsequently, for the real-time polymerase chain reaction (PCR) analysis, a real-time PCR kit containing SYBR Green master mix was utilized. In addition to this, specific primers were employed for both the target genes of interest (such as NF-кB, iNOS, COX-2, TNF-α) and a housekeeping gene, typically glyceraldehyde-3-phosphate dehydrogenase (GAPDH). This approach allowed for the quantification of gene expression levels in a precise and highly controlled manner. Gene expression levels were calculated using the ΔΔCt method for relative quantification of gene expression. The ΔCt was calculated by subtracting the Ct of housekeeping gene GAPDH from the Ct of the mRNA of interested gene. The LPS-BJ-1 ΔΔCt was calculated by subtracting the ΔCt of the interested gene (BJ-1 cells before LPS treated) from the ΔCt of each interested gene (BJ-1 cells post LPS treated). The PVA/AH LPS-BJ-1 ΔΔCt was calculated by subtracting the ΔCt of the interested gene (LPS-BJ-1 cells before PVA/AH treated) from the ΔCt of each interested gene (LPS-BJ-1 cells post PVA/AH treated).The formula 2^−ΔΔCt^ was used to get the fold change (39–42).

### 2.16 Statistical analyzing data

The statistical significance level was set at a p-value of less than 0.001. The data were presented as the mean plus or minus the standard deviation. In order to assess the discrepancies in the average values across the examined groups, a statistical analysis was performed employing two methodologies: Duncan’s analysis and one-way analysis of variance (ANOVA). The IC50 values for the *in vitro* antioxidant tests were assessed using GraphPad software version 8, and the analysis was conducted using SPSS program version 22.

## 3 Results

### 3.1 Physiochemical characterization of PVA/AH

#### 3.1.1 FTIR analysis

**Figure 1:**
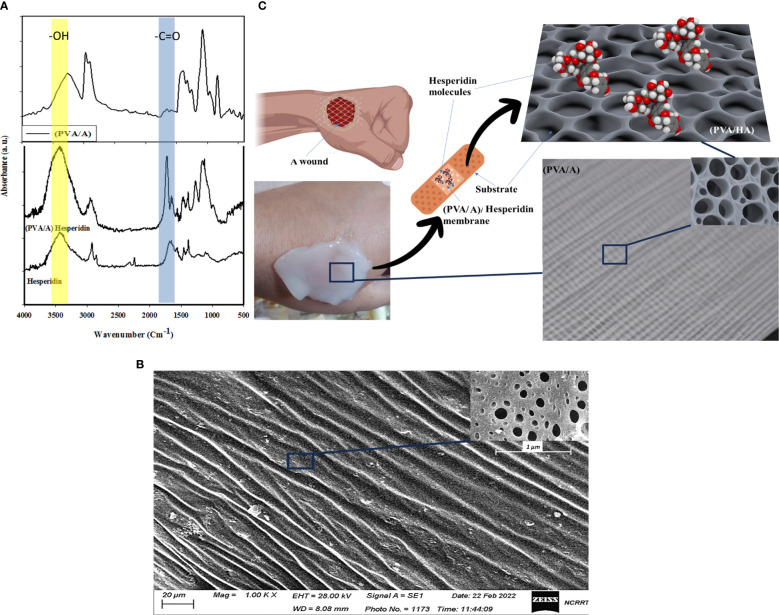
Show FTIR of H, PVA/A, and PVA/AH membrane **(A)**, SEM image of (PVA/AH) membrane **(B)** and a simulation of the loaded H and their release for wound dressing with (PVA/AH) membrane **(C)**.

**Figure 1A** displays the Fourier-transform infrared (FTIR) spectrum of a blend consisting of Polyvinyl Alcohol (PVA) and Sodium Alginate. In the spectrum, the peak at 3269 cm^-1^ is assigned to the O-H stretching vibration of hydroxyl groups, which are present in both PVA and Sodium Alginate. The peak observed at 1663 cm^-1^ corresponds to the C=O (carbonyl) stretching vibration, indicating the presence of carbonyl groups. These carbonyl groups may originate from acetyl groups in Sodium Alginate. This FTIR analysis provides valuable information about the chemical composition and functional groups present in the polymer blend. The peak at 1402 cm^-1^ might be related to the C-H bending vibrations in the CH_2_ and CH_3_ groups present in both PVA and Sodium Alginate. The peak at 1083 cm^-1^ could be due to the C-O stretching vibration in both the ether linkages of PVA and the glycosidic linkages in Sodium Alginate. The peak observed at 846 cm^-1^ in the FTIR spectrum corresponds to the C-H out-of-plane bending vibrations in the CH_2_ groups present in both Polyvinyl Alcohol (PVA) and Sodium Alginate. The peak at 647 cm^-1^ might be related to the C-H out-of-plane bending vibrations in the CH_3_ groups present in both PVA and Sodium Alginate. Also, **Figure 1A** shows the catachrestic FTIR peaks of H molecule such as the broad peak at 3437 cm^-1^ corresponds to hydroxyl (OH) group stretching, which is characteristic of the flavonoid’s polyphenolic structure. At 1689 cm^-1^, the carbonyl (C=O) stretching vibrations are evident. Additionally, two significant peaks occur at 1570 cm^-1^ and 1451 cm^-1^, which can be attributed to the presence of aromatic C=C stretching vibrations in the H molecule. The peaks at 1377 cm^-1^, 1214 cm^-1^, and 1103 cm^-1^ signify C-O stretching and C-C vibrations, indicating the presence of various functional groups within H. A shift from 3437 cm^-1^ to 3422 cm^-1^ in the hydroxyl (OH) group stretching vibrations suggests the formation of hydrogen bonds between H and the polymers. The appearance of a new peak at 1733 cm^-1^ signifies ester carbonyl (C=O) stretching vibrations originating from the polymer matrix. Furthermore, the peak at 1273 cm^-1^ corresponds to C-O stretching, demonstrating the influence of H on the polymer structure. At 1155 cm^-1^, a shift in the peak position denotes potential interactions involving C-O or C-C stretching vibrations. These spectral changes affirm the incorporation of H within the PVA and sodium alginate matrix, highlighting the establishment of new interactions and chemical bonds by inter-molecular H bonds.

#### 3.1.2 SEM analysis

**Figure 1B** shows an SEM image (scale 20 μm) of a membrane composed of PVA and A. The image reveals the membrane has a porous surface structure, with pores/openings visible throughout the surface of the membrane material. The presence of a porous surface indicates the PVA/A membrane is a dense surface. Rather, it has a porous network/architecture at the micrometer scale as seen under SEM magnification. A porous surface could be beneficial for applications requiring the transport of molecules, liquids, gases, etc. through the material. In the case of a wound dressing, a porous membrane could allow for controlled release of active ingredients like H from the dressing material into the wound site over time. As H diffuses out, new H from inside the pores could continuously replace it, providing sustained release properties. The pores may also support fluid transport into and out of the dressing, wicking away excess wound exudate while still keeping the wound moist. In summary, the clear porosity observed in the SEM image suggests the PVA/A membrane has a structure that could be well-suited for wound dressing applications requiring controlled release and fluid handling capabilities. **Figure 1C** presents a simulation indicating the potential application of the (PVA/A) membrane as a drug delivery platform for H, which is an anti-inflammatory drug used for treating wound infections. The simulation process involves generating a stable configuration of the (PVA/A) membrane interacting with H. The interaction is primarily achieved through hydrogen bonding between the oxygen atom on the hydroxyl group of PVA and A, and the hydrogen atom of the carboxylic group on H. The (PVA/A) membrane is being considered as a substrate for delivering H, an anti-inflammatory drug. This suggests that the porous nature of the membrane, along with its potential to interact with H, could enable controlled release of the drug over time. The simulation process in **Figure 1C** involves finding a stable configuration for the interaction between the (PVA/A) membrane and Hesperidin. This typically involves computational methods that predict how molecules would interact and arrange themselves based on their chemical properties and forces. Hydrogen bonding is a type of intermolecular interaction that occurs between a hydrogen atom and an electronegative atom (usually oxygen, nitrogen, or fluorine) on another molecule. In this case, the oxygen atom on the hydroxyl group of both PVA and A is forming hydrogen bonds with the hydrogen atom of the carbonyl group on H. Hydrogen bonding is a crucial force in molecular interactions and can affect how molecules bind and interact with each other. The hydrogen bonding interaction described in the simulation suggests that the (PVA/A) membrane could effectively retain H through these bonds. However, under certain conditions (such as changes in pH, temperature, or the presence of specific enzymes), the hydrogen bonds could weaken, facilitating the release of H from the membrane. This controlled release mechanism is advantageous for achieving a sustained therapeutic effect over time. The simulation in

#### 3.1.3 Simulation of drug delivery platform for hesperidin

**Figure 1C** provided valuable insights into the potential interaction between the (PVA/A) membrane and H, supporting the membrane’s suitability as a drug delivery system for the anti-inflammatory drug. However, it’s important to note that simulations are theoretical models and need to be validated through experimental studies before any practical applications can be considered. Additional information about the (PVA/A) wound dressings prepared by the gamma irradiation process and their relevant properties should be provides. The (PVA/A) wound dressings have a substantial thickness of approximately 3 mm, which can be advantageous for wound care applications, providing a substantial barrier and cushioning. SEM analysis has confirmed the presence of porosities in these dressings, which can facilitate breathability and interaction with the wound environment. The WVTR values for the (PVA/A) wound dressings, prepared via gamma irradiation, are within the range of 1012–1141 g/m² per day. These rates indicate the ability of the dressings to allow the passage of water vapor, which is essential for maintaining a suitable microenvironment at the wound site. Interestingly, the water permeation rates of the (PVA/A) wound dressings do not appear to be significantly affected by variations in evaporation time during the drying step. This suggests that the dressings maintain consistent water permeation properties regardless of the drying conditions. The oxygen permeation rates for the (PVA/A) wound dressings fall within the range of 430–1394 liters/m² per day. These values indicate the ability of the dressings to allow the passage of oxygen, which is essential for wound healing and tissue regeneration. The (PVA/A) wound dressings exhibit water uptake capacity of approximately 33%. This capacity suggests that the dressings can effectively absorb and retain moisture, potentially promoting a favorable wound healing environment. It is noteworthy that the swelling process does not lead to deformation or dissolution of the (PVA/A) wound dressings. This indicates the structural stability of the dressings even when they undergo significant swelling, which is essential for maintaining contact with the wound surface. Overall, the (PVA/A) wound dressings prepared by the gamma irradiation process demonstrate promising characteristics for wound care applications. Their ability to control water vapor and oxygen permeation, coupled with their substantial water uptake capacity and structural integrity during swelling, suggests their potential effectiveness in supporting the wound healing process.

### 3.2 pH-sensitivity of (PVA/A) hydrogel biomaterial

**Figure 2:**
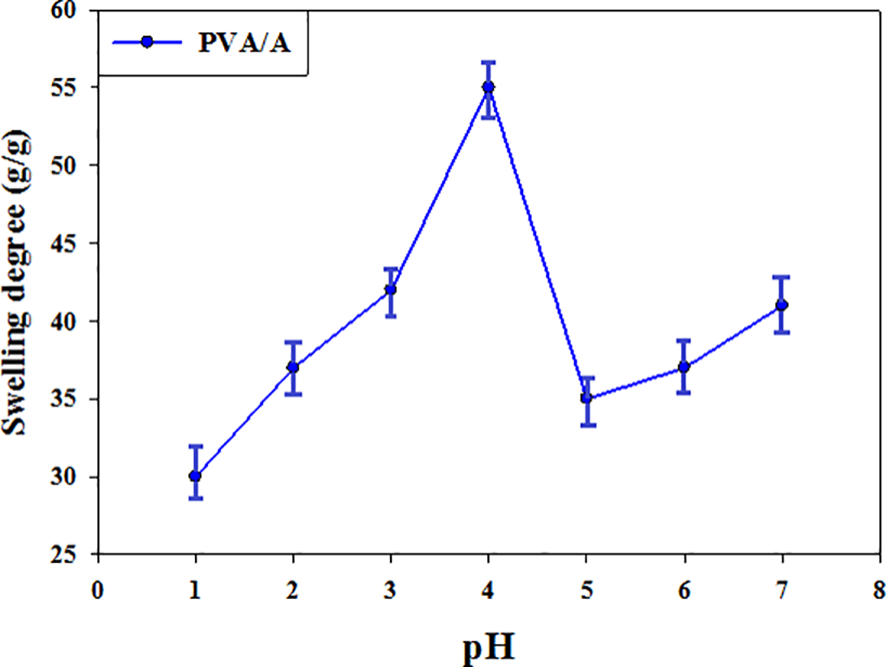
The pH swelling sensitivity of (PVA/A) hydrogel biomaterials.

The pH-swelling dependence of the crosslinked (PVA/A) hydrogel biomaterial was investigated, and the results are presented in **Figure 2**. The hydrogel demonstrated pH sensitivity due to the presence of carboxyl functional groups (-COOH) in sodium alginate (A). The swelling degree of the hydrogel varied with the pH, covering a range from 1 to 7. At pH 1, the swelling degree reached a minimum of 30 g/g. This can be attributed to the -COOH groups being predominantly undissociated at this acidic pH. However, as the pH increased from 2 to 4, the dissociation of the COOH groups increased. This led to an enhancement in the swelling degree, with values of 37 g/g, 42 g/g, and 55 g/g at pH 2, 3, and 4, respectively. Of particular interest, the maximum swelling was observed at pH 3 and pH 4, corresponding to the pKa values of 3.2 and 4 for guluronic acid and mannuronic acid, respectively, in alginate molecules. At these pH values, half -COOH groups were dissociated, resulting in maximum swelling behavior of the hydrogel. As the pH further increased to 5, the -COOH groups started to deprotonate and were substituted with Na+ ions. This substitution decreased the swelling degree to 35 g/g. At pH levels of 6 and 7, the swelling degree exhibited a further rise to 37 g/g and 41 g/g, respectively. The observed rise in levels may be ascribed to an elevation in sodium (Na+) ions at higher pH (43, 44). The presence of more Na+ ions decreased the intermolecular hydrogen bonds between A and PVA molecules, causing an increase in the porous size and water absorbance from pH 6 up to pH 7. These findings demonstrate the pH-responsive behavior of the (PVA/A) hydrogel biomaterial, with its swelling degree being influenced by the dissociation of carboxyl groups and the presence of Na+ ions. Understanding the pH-swelling relationship is important for tailoring the properties and applications of the hydrogel in various biomedical and pharmaceutical fields.

### 3.3 Mechanical property of (PVA/A) hydrogel biomaterials

**Figure 3:**
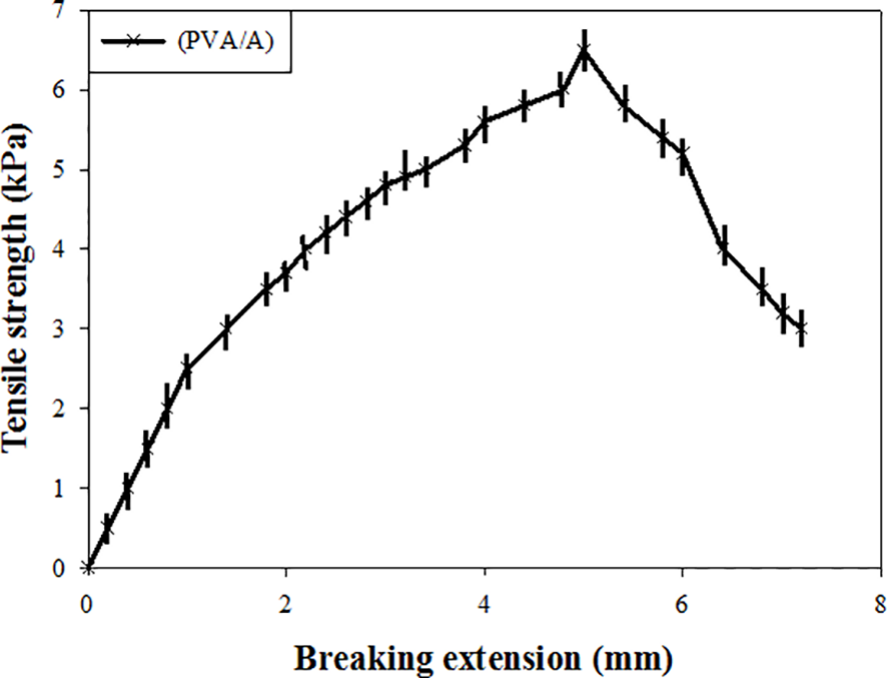
The tensile property of the wet (PVA/A) hydrogel biomaterial dressing.

The tensile property of the wet (PVA/A) hydrogel biomaterial dressing were evaluated. The tensile strength of the dressing was determined to be 6.5 KPa, indicating its ability to withstand applied forces without breaking. Additionally, the breaking extension of the dressing was found to be within the limit of 6* mm*, indicating its suitability as a wound dressing. These results demonstrate that the wet treatment had a significant effect on the tensile strength of the dressings. The dressing exhibited sufficient tensile strength to withstand mechanical stresses typically encountered during application and use as a wound dressing. Moreover, the breaking extension of the dressings was appropriate, suggesting they can be effectively used for wound healing purposes. The tensile properties of the (PVA/A) hydrogel biomaterial dressing play a crucial role in determining its mechanical integrity and ability to conform to different wound types. The reported tensile strength and breaking extension values indicate that the dressing possesses desirable mechanical properties for wound dressing applications, providing support and protection to promote the healing process (**Figure 3**).

### 3.4 Cytotoxicity of poly (PVA/A) and poly (PVA/AH) hydrogels on human normal skin cell line (BJ-1)

**Table 2** displays the survival of BJ-1 cells on poly (PVA/A) and (PVA/AH) hydrogels. After 48 hours in culture, both samples demonstrated signs of active growth in BJ-1 cells. The IC50 value for (PVA/A) was determined to be 314.1 µg/mL, while it was 467 µg/mL for (PVA/AH). These results indicate that both hydrogels are non-toxic to BJ-1 cells. Furthermore, these findings suggest that the hydrogels have the potential to facilitate the extended release and transport of H without adversely affecting cell viability.

**Table 2:** The cell viability on poly (PVA/A) and poly (PVA/AH) hydrogels.

Cell viability of PVA/A hydrogel			
Concentration µg/mL	Mean ± SD	Viability%	IC50 µg/mL
0	0.704 ± 0.007	100	314.1
6.5	0.696 ± 0.003	98.8	
12.5	0.678 ± 0.007	96.3	
25	0.661 ± 0.008	93.9	
50	0.647 ± 0.004	91.9	
100	0.553 ± 0.010	78.6	
Cell viability of PVA/AH hydrogel			
Concentration µg/mL	Mean ± SD	Viability%	IC_50_ µg/mL
0	0.704 ± 0.007	100	467.4
6.5	0.674 ± 0.003	95.8	
12.5	0.656 ± 0.011	93.2	
25	0.638 ± 0.009	90.7	
50	0.583 ± 0.011	82.9	
100	0.546 ± 0.005	77. 6	

### 3.5 Effect of poly (PVA/A) and poly (PVA/AH) hydrogels on DPPH radical scavenging activity

**Figure 4:**
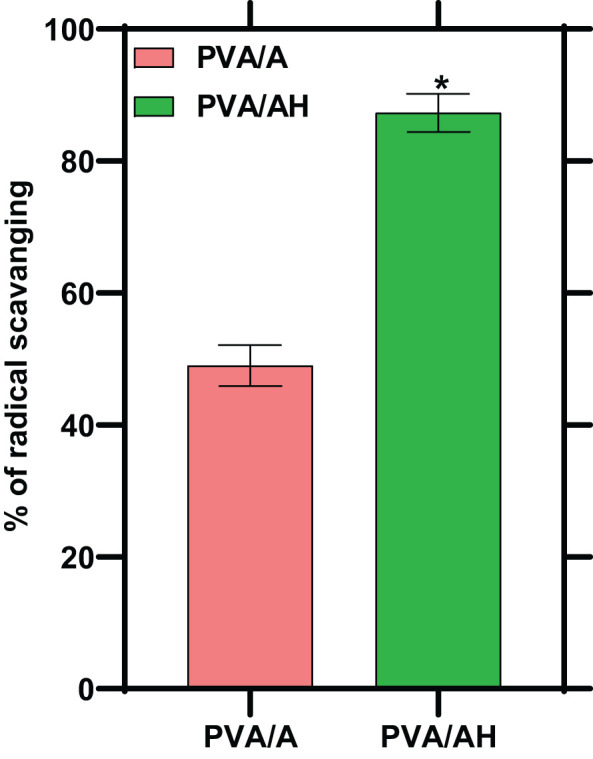
Radical scavenging activity of PVA/AH (*) significantly (p< 0.001) higher than PVA/A.

Oxidation can lead to the overproduction of free radicals, initiating a cascade of events that can cause damage to cells. An antioxidant is a substance that inhibits oxidation. In the present study, the antioxidant activity of the hydrogels was assessed by measuring their free radical-scavenging activity. This activity indicates the hydrogels’ ability to neutralize and counteract the harmful effects of free radicals, which are highly reactive molecules involved in oxidative stress and cellular damage. As shown in **Figure 4**. The % of radical scavenging was 49% for poly (PVA/A) hydrogel and 87% for poly (PVA/AH) hydrogel, indicating that the presence of H potentiate the antioxidant activity of the hydrogel.

### 3.6 Anti-inflammatory activity of PVA/A and PVA/AH hydrogels

**Figure 5:**
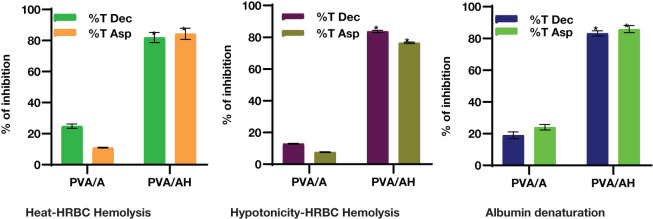
*In vitro* anti-inflammatory action of PVA/AH (*) significantly (p< 0.001) higher than PVA/A on HRBCs..

The employed an *in vitro* experiment to evaluate the anti-inflammatory characteristics of PVA/A and PVA/AH hydrogels. The first topic to be discussed is heat-induced hemolysis of human red blood cells (HRBC) and the subsequent stabilization of the cell membrane. Additionally, the phenomenon of HRBC hemolysis caused by hypotonic conditions will be examined. Lastly, the process of protein denaturation will be addressed (**Figure 5**).

Lysosomal lysis is a process that can occur during inflammation, resulting in the release of lysosomal enzymes. These enzymes can contribute to various pathological conditions and tissue damage. The assessment of the anti-inflammatory action of PVA/A and PVA/AH hydrogels can be performed by inhibiting the lysis of red blood cell membranes generated by heat and hypotonicity, as these membranes exhibit similar functionality to lysosomal membranes.

#### 3.6.1 Heat-Induced Hemolysis of HRBC

When heat increased cell membrane is ruptured due to the excessive accumulation of fluids within the cellular environment. As shown in **Figure 2**, compared to diclophenac (Dic) and aspirin (ASP), the % of inhibition against heat-induced hemolysis of RBCs was 24.83 and 10.97% for PVA/A hydrogel and 81.97 and 84.34% for PVA/AH hydrogel, respectively, indicating that PVA/AH hydrogel may play a significant role in the protection of membranes.

#### 3.6.2 Hypotonicity- Induced Hemolysis of HRBC

Hypotonic solutions can lead to an excess accumulation of fluid inside cells, which can result in the rupture of the red blood cell (RBC) membrane. This phenomenon is known as hemolysis. When RBCs burst, they release serum proteins and fluids into the surrounding tissue, which can contribute to tissue damage and inflammation. Membrane stabilization is a process or property that helps prevent this rupture or hemolysis. Compared to diclophenac and aspirin, the % of inhibition of was 12.89 and 7.67% for PVA/A hydrogel and 83.68 and 76.48% for PVA/AH hydrogel, respectively. The PVA/AH hydrogel has the potential to preserve the integrity of red blood cells (RBCs) by impeding the release of lytic enzymes and other inflammatory mediators.

#### 3.6.3 Protein denaturation test

Protein denaturation is indeed one of the well-known and primary causes of inflammation. Compared to diclophenac and aspirin, the % of inhibition of protein denaturation was 18.95 and 23.98% for PVA/A hydrogel, respectively and 83.17 and 85.8% for PVA/AH hydrogel, respectively indicating that H may play a significant role in preventing protein denaturation and contribute to HRBC membrane stability.

### 3.7 PVA/AH hydrogel influences ROS, NO, and MPO levels.

**Figure 6:**
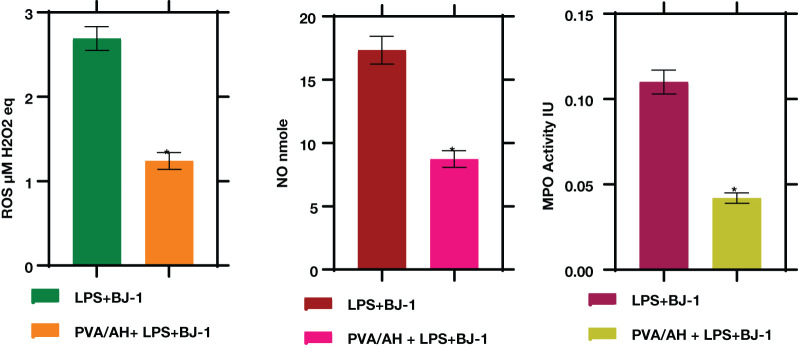
Action of PVA/AH hydrogel on oxidative markers. (*) significant (*p*< 0.001).

In LPS-treated BJ-1 cells incubated with PVA/AH hydrogel for 48 hours, the MPO activity were significantly lower (P<0.001) (0.042± 0.003) than its value (0.11± 0.007) in the LPS-treated BJ-1 cells. The levels of ROS (4.42 ± 0.23) and NO (17.33 ± 1.1) in LPS-treated BJ-1 cells were significantly lower (P<0.001) (1.88 ± 0.12) and (8.73 ± 0.65) in the LPS-treated BJ-1 cells incubated with PVA/AH (**Figure 6**).

### 3.8 Effect of PVA/AH hydrogel on antioxidant status (GPX and SOD activities and TAC)

**Figure 7:**
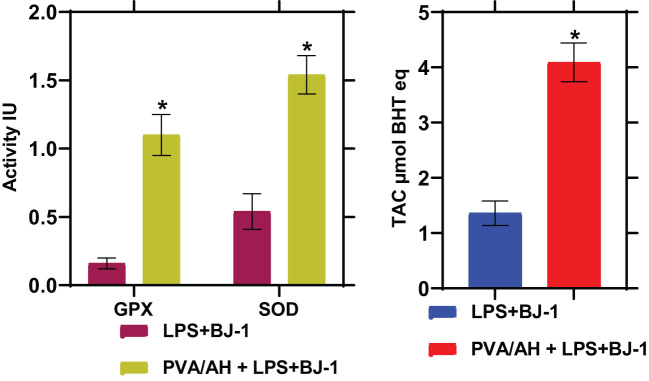
Action of PVA/AH hydrogel on GPx and SOD activities and TAC. (*) significant (*p*< 0.001).

In LPS-treated BJ-1 cells incubated with PVA/AH hydrogel for 48 hours, the GPX and SOD activities were significantly higher (P<0.001) (1.1± 0.15) and (1.54± 0.14), respectively than their corresponding level (0.16± 0.04) and (0.54± 0.13), in the LPS-treated BJ-1 cells. Furthermore, the TAC was higher (4.09± 0.35) for PVA/AH hydrogel, compared to (1.36 ± 0.22) in case of LPS-treated BJ-1 cells (**Figure 7**).

### 3.9 Effect of PVA/AH hydrogel on gene expression of inflammatory markers, TNF-α, NFκB, INOS, and COX-2

**Figure 8:**
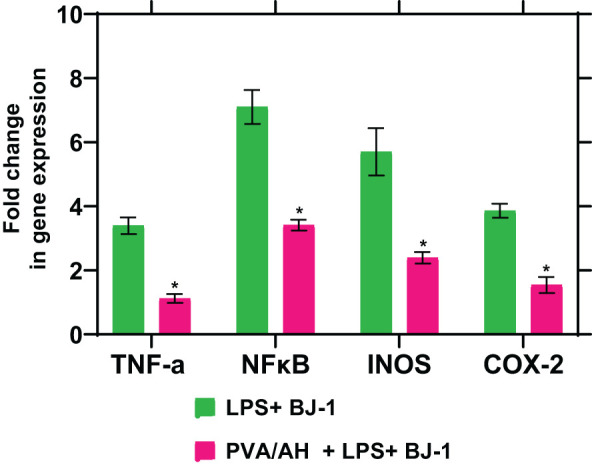
Action of PVA/AH hydrogel on the expression of TNF-α, NFκB, iNOS, and COX-2. (*) significant (*p*< 0.001).

When LPS-treated BJ-1 cells were incubated with PVA/AH the fold change in gene expression for TNFα, NFκβ, iNOS and COX2 was 1.12± 0.14, 3.41± 0.17, 2.39± 0.18, and 1.54± 0.25 respectively, compared to 3.39± 0.26, 7.1± 0.53, 5.7± 0.74 and 3.86± 0.22 for LPS-treated BJ-1 cells (**Figure 8**). The results suggest that H may exert anti-inflammatory effect by inhibiting the expression of pro-inflammatory mediators TNFα, NFκB, iNOS and COX2

## 4 Discussion

The skin, being the body’s largest organ, is susceptible to various diseases and injuries, just like any other organ in the body. It serves as a critical interface between the internal environment of the body and the external world. This exposure to the environment makes the skin more vulnerable to external factors, including physical, chemical, and microbial stress One specific concern mentioned is the potential for skin injuries resulting from acute radiation exposure. Radiation exposure can indeed lead to skin wounds and burn injuries The study explores methods to address and treat skin injuries caused by radiation exposure, with a particular focus on the potential benefits of the PVA/AH hydrogel in wound healing and inflammation control (45). Irradiation causes multiple negative effects on the wound healing processes that include inhibition of connective tissue proliferation, formation and maturation of granulation tissue, and transcription of collagen mRNAs (45). Also, delayed skin healing is due to disturbed angiogenesis, prolonged inflammation phase, damage of extracellular matrix (ECM), elevated oxidative stress, and increased pro-inflammatory cytokines (9, 46).

The study addresses a significant medical challenge related to skin wound healing, particularly in individuals with diabetes and those undergoing radiotherapy treatment. Both of these groups often face difficulties in wound healing, and finding effective wound management strategies is crucial. In this study, a hydrogel was developed using a combination of polyvinyl alcohol (PVA) and sodium alginate (A). This hydrogel was created through a process involving gamma radiation and was enriched with H, a natural compound with potential therapeutic properties. The study conducted cytotoxicity tests on both the hydrogels (PVA/A and PVA/AH) and found that they were non-toxic. This is a crucial safety aspect when considering medical applications. Additionally, the study observed that the PVA/AH hydrogel exhibited a higher radical scavenging activity compared to the PVA/A hydrogel. This suggests that the presence of H in the hydrogel enhances its antioxidant capabilities. The results of this study indicate that the PVA/AH hydrogel, with its combination of materials and the inclusion of H, could have potential benefits for wound healing in individuals with skin wounds, particularly in the challenging contexts of diabetes and radiotherapy. It may offer antioxidant and tissue-protective effects that could aid in the healing process.

Oxidative stress can impede wound healing (19, 47). The results of extensive studies showed that pretreatment of keratinocytes (mouse skin) with H significantly reduced radiation-induced oxidative stress (48), lipid peroxidation, while increasing the expression levels of antioxidant enzymes (20, 49). Moreover, H can prevent the radiation induced reductions in antioxidant capacity and the increase in myeloperoxidase activity (20). The flavonoid H has significant antioxidant and antibacterial properties ideally repairing skin injuries. In the current study, the activity of the antioxidant enzymes GPX and SOD and the level of TAC was higher for PVA/AH hydrogel compared to their levels in LPS-treated BJ-1 cells. Furthermore, the level of ROS, and NO and the activity of MPO was lower for PVA/AH hydrogel compared to their levels in LPS-treated BJ-1 cells. The study results suggest that the antioxidant activity of the PVA/AH hydrogel extends beyond its capacity to scavenge free radicals. Specifically, the hydrogel appears to enhance the antioxidant defenses within cells. This indicates that the hydrogel, possibly due to the presence of H, not only directly counteracts oxidative stress by neutralizing free radicals but also stimulates the cellular mechanisms responsible for antioxidant protection (10, 45, 46).

Nitric oxide (NO) is indeed an important inflammatory mediator and is frequently used as a biomarker to assess inflammatory responses. In the context of inflammation, NO is often produced by immune cells as a signaling molecule and can contribute to the inflammatory process. Therefore, reducing NO levels can be an indicator of anti-inflammatory activity The study’s *in vitro* experiment demonstrated that the addition of H to the culture medium resulted in lower nitrite levels in cells treated with lipopolysaccharide (LPS) (50). In the current study, the level of iNOS and NO was lower for PVA/AH hydrogel compared to LPS-treated BJ-1 cells, demonstrating the anti-inflammatory properties of H (10, 15, 45).

*In vitro* anti-inflammatory studies showed that PVA/AH hydrogel inhibited heat-induced HRBC hemolysis, hypotonicity-induced hemolysis, and egg albumin denaturation better than PVA/A. The results indicate that H may play a significant anti-inflammatory role (51, 52) in membrane stabilization of HRBC. In addition, since lysosomal membrane and HRBC membrane have the same components, thus H may have a significant role in the integrity of cell membrane damage (53, 54), and inhibit lysosomal lysis during inflammation and the release of enzymes (55, 56).

Cytokines play a central role in regulating the inflammatory response in the body. They are signaling molecules that are produced by various cells, including immune cells, to coordinate and modulate the immune and inflammatory processes. Cytokines can stimulate, recruit, and induce the proliferation of immune cells, thereby orchestrating the body’s response to infections, injuries, and other immune challenges. Cytokines includes interleukins (IL), chemokines, interferons, and tumor necrosis factors (TNF) (57). Inflammatory mediators, include proteins, peptides, glycoproteins, cytokines (TNF), prostaglandins, leukotrienes, nitric oxide, and oxygen free radicals (58). Hydrogels, such as the PVA/AH hydrogel mentioned in the study, have shown promise in generating anti-inflammatory effects by modulating the production of pro-inflammatory mediators. Here’s how hydrogels can contribute to anti-inflammatory benefits (57). In the current study, the fold change in gene expression for TNFα, NFKβ, iNOS and COX2 with PVA/AH was lower, compared to their values for LPS-treated BJ-1 cells. The results are in accordance that H protects against inflammation (59). The studies demonstrating the preventive effect of topical H on radiation-induced elevations in cutaneous cytokine expression highlight the potential of H as a protective agent against radiation-induced skin damage. These findings suggest that when applied topically to the skin before radiation exposure, H may mitigate the inflammatory response and the release of cytokines associated with radiation-induced skin injury. This protective action may help reduce the severity of radiation-induced skin damage and associated symptoms (48, 60). Also, in both rat skin and human keratinocyte cultures, H could decrease the expression of TNFα, NF-ϰB, and cyclooxygenase-2 (COX-2) (18, 60) protein and iNOS mRNA levels (50, 61).

The topical administration of H gel has demonstrated effective results in the treatment of wounds (10, 62, 63). The evaluation of H for its wound healing activity in irradiated mice is a significant research area with potential clinical implications (64). The study demonstrates that in γ-irradiated rats the topical administration of H accelerated the healing of wounds by increasing the collagen, hexosamine, DNA, and nitric oxide syntheses and increasing the densities of fibroblasts and blood vessels in the regenerating wounds (15, 45). Also, H accelerates angiogenesis (65), and extends an antioxidant effect through scavenging free radicals or via stimulating cellular antioxidant enzyme systems (9).

## 5 Conclusion

The study involved the synthesis of a PVA/A hydrogel designed for loading and controlled release of H. The hydrogel’s properties and interactions were characterized and confirmed using FTIR (Fourier-Transform Infrared Spectroscopy). The prepared PVA/AH hydrogel membrane was found to be non-toxic, and its *in vitro* anti-inflammatory activity was assessed using the Human Normal Skin cell line (BJ-1). The study examined the impact of the hydrogel on a range of necroinflammatory markers. The anti-inflammatory potential of PVA/AH hydrogels was evaluated using methods such as heat-induced membrane stabilization, hypotonicity-induced HRBC membrane stabilization, and egg albumin denaturation. The results of the heat-induced anti-inflammatory test demonstrated that the hydrogels PVA/A exhibited protective effects on the human erythrocyte membrane, reducing heat-induced lysis by 24.83% and 10.97% respectively. Additionally, the hydrogel PVA/AH showed even greater protection, with reductions in heat-induced lysis of 81.97% and 84.34% compared to the reference drugs Dic. and Asp., respectively, at a concentration of 250 μg/ml.

Hypotonicity-induced anti-inflammatory test showed that hydrogels PVA/A protected the human erythrocyte membrane against lysis (12.89 and 7.67%) and PVA/AH (83.68 and 76.48%) as compared, respectively, to 250 μg/ml Dic. and Asp. Egg albumin denaturation also assessed hydrogels as compared, respectively, to 250 μg/ml Dic. and Asp., PVA/A (18.95 and 23.98%) and PVA/AH (83.17 and 85.8%). The DPPH activity of PVA/A and PVA/AH hydrogels was 49% and 87%, respectively. PVA/AH substantially decreased ROS, and NO, and by 57.5 and 49.6%, respectively, and raised TAC by 201% in LPS-stimulated BJ-1 cells. The activities of GPX and SOD increased by 587% and 185%, respectively. In contrast, MPO activity decreased by 61.8%. PVA/AH treatment resulted in the downregulation of TNFα, NFκB, iNOS, and COX2 by 67%, 52%, 58%, and 60%, respectively, as compared to BJ-1 cells activated with LPS. The findings of our study indicate that all hydrogels exhibited a lack of toxicity. The IC_50_ for PVA/A was 314.1 µg/mL and 467 µg/mL for PVA/AH µg/ml, respectively. Given the antioxidants, and anti-inflammatory effects of H, we hypothesize that the combination of PAV/A hydrogel loaded with H PAV/AH could have a synergetic effect to accelerate wound healing.

## Data availability statement

The raw data supporting the conclusions of this article will be made available by the authors, without undue reservation.

## Ethics statement

Ethical approval was not required for the studies on humans in accordance with the local legislation and institutional requirements because only commercially available established cell lines were used. Ethical approval was not required for the studies on animals in accordance with the local legislation and institutional requirements because only commercially available established cell lines were used.

## Author contributions

AH: Writing – review & editing, Writing – original draft, Visualization, Validation, Supervision, Software, Project administration, Methodology, Investigation, Formal analysis, Data curation, Conceptualization. AK: Writing – review & editing, Writing – original draft, Visualization, Validation, Software, Methodology, Investigation, Formal analysis, Data curation. MA-M: Writing – review & editing, Visualization, Validation, Supervision, Resources, Project administration, Methodology, Investigation, Formal analysis, Data curation, Conceptualization. ME-T: Writing – original draft, Validation, Methodology, Investigation, Formal analysis. DA-S: Writing – original draft, Validation, Methodology, Investigation, Formal analysis. SSM: Writing – original draft, Validation, Methodology, Investigation, Formal analysis. MG: Writing – original draft, Validation, Methodology, Investigation, Formal analysis. AE: Writing – original draft, Validation, Methodology, Investigation, Formal analysis. SE-H: Writing – original draft, Validation, Methodology, Investigation, Formal analysis. SH: Writing – original draft, Validation, Methodology, Investigation, Formal analysis. SM: Writing – original draft, Validation, Methodology, Investigation, Formal analysis. AR: Writing – original draft, Validation, Methodology, Investigation, Formal analysis. HS: Writing – original draft, Visualization, Validation, Resources, Methodology, Investigation, Funding acquisition, Formal analysis, Data curation.
